# Defining outcome measures in juvenile idiopathic arthritis associated uveitis by a systematic review analysis: do we need a consensus?

**DOI:** 10.1186/s12969-019-0330-9

**Published:** 2019-07-11

**Authors:** Greta Mastrangelo, Ivan Foeldvari, Jordi Anton, Gabriele Simonini

**Affiliations:** 1 Rheumatology Unit, Anna Meyer Children’s Hospital, Pediatric Section-NEUROFARBA Department, University of Florence, Florence, Italy; 2 Centre for Pediatric and Adolescence Rheumatology, An der Schön Klinik, Hamburg, Eilbek Germany; 3 Pediatric Rheumatology, Hospital Sant Joan de Déu, Barcelona, Spain

**Keywords:** Juvenile idiopathic arthritis, Uveitis, Outcome

## Abstract

**Background:**

Juvenile Idiopathic Arthritis associated Uveitis (JIA-U) represents its most frequent extra-articular manifestation and the main cause of childhood uveitis in in developed countries. The broad variety of outcome measures utilized makes the comparison of the disease course, risk for complications, impairment in visual function, and responses to treatment quite difficult. Our aim was to summarize evidence regarding the current availability of outcome measures in JIA-U.

**Methods:**

A systematic review between January 2000 and December 2018 was performed to identify studies investigating outcome measures used in JIA-U.

**Results:**

The initial search identified 8254 articles of which 89 were potentially eligible. After the full text revision, a total of 27 studies, including 2 RCTs, were included. Among these studies 12 outcome measures for JIA-U use have been identified (grade of cells in the AC, grade of flare in the AC, VA, amblyopia, structural complications, use and sparing of oral corticosteroids and immunosuppressive drugs, surgery requirement, biomarkers, bilateral disease, JIA persistence, quality of life assessments, uveitis subtype). As regards primary outcome measures, 44% among studies included one or more variables related to disease activity (i.e. grade of flare, grade of cells); 56% included visual function performance (i.e. visual acuity); 68% (17/25) included one or more variables of disease-associated tissue damage or complications (i.e. cataract, amblyopia); 24% included disease features (i.e. bilateral disease; uveitis subtype); 44% included laboratory features (i.e. biomarkers); 8% included JIA features (i.e. persistence of disease); 12% included quality of life (i.e. EYE-Q); 44% included management (i.e. use and sparing of oral corticosteroids and other immunosuppressive drugs; surgery requirement).

**Conclusions:**

Our systematic review surveys the heterogeneity around outcome measures related to JIA-U in children, even in RCTs. It does not provide the solution to overcome the heterogeneity in uveitis studies, but it does provide an estimate of the scale of the problems and provides data to inform this important debate; highlighting the requirement to obtain a new consensus regarding a common approach to identify suitable and efficient outcome measures in JIA-U.

## Background

Juvenile Idiopathic Arthritis associated Uveitis (JIA-U), with an estimated incidence at 8.3%, represents its most frequent extra-articular manifestation and is the main cause of uveitis in pediatrics in developed countries [1]. JIA-U is a serious and disabling sight-threatening disease, accounting for up to 10% of pathologies leading to blindness. Patients with JIA develop uveitis in approximately 50 and 90% within 3 months and 4 years, respectively, after the diagnosis of arthritis. Uveitis precedes the onset of arthritis in 2–7% of patients. Uveitis, usually affecting anterior chamber, is asymptomatic in the majority of JIA children, aside entesitis-related arthritis and late onset psoriatic arthritis JIA type, which may instead experience symptomatic uvea involvement. Any way, periodic ophthalmologic examination is mandatory required for screening [2]. In JIA-U care, to define related and unified outcome measures are for sure quintessential to assess the effect of the treatment. Observations published worldwide on the outcomes of JIA-U are surprisingly diverse and different. The broad variety of outcome measures utilized makes the comparison of the disease course, risk for structural complications, levels of impairment in visual function, and responses to treatment quite difficult.

Of note, standardizing outcomes allows data comparison, and a core set of outcomes, agreed upon by both different involved researchers and patients, provides a common focus for interventional studies. Disease-specific and universally agreed upon outcomes are likely to reduce selective reporting and reporting bias [3].

The Standardization of Uveitis Nomenclature (SUN) Working Group has provided a standardized nomenclature of uveitis, inflammation grading, and outcome measures [4]. This instrument is of importance for the comparison of outcomes and trials, and for the optimal design of future trials.

Since specific outcome measures have not been established for JIA-U till 2010, a multinational interdisciplinary working group of ophthalmologists and paediatric rheumatologists gathered to consider this issue for the first time and published the first JIA-U specific proposal [5].

They reviewed the recommendations of the SUN Working Group with special consideration given to their applicability for reporting clinical outcomes in studies of JIA-U. The proposed outcome measures were: measurement of uveitis activity (grade of cells in the AC, grade of flare in the AC, number of visits with active uveitis), visual acuity (measurement of visual acuity, amblyopia), development of structural complications (synechiae formation, cataract formation, macular edema, epiretinal membrane formation, band keratopathy, ocular hypertension and glaucoma, ocular hypotony, vitreous haze and cells), quality of life assessments, sparing of corticosteroids and other immunosuppressive drugs, surgery requirement, biomarkers.

To our knowledge no others studies have been conducted on this issue. Aim of our study was to summarize evidence regarding the current availability of outcome measures in JIA-U. We aimed to provide a complete, exhaustive, systematic literature review to describe the cutting edge of knowledge about outcome measures for use in JIA-U. The literature relating to outcome measures used in JIA-U studies in childhood and adolescence was reviewed.

## Methods

A systematic review was conducted to identify the existing published evidence concerning the use or the proposed use of outcome measures in JIA-U. The results are shown according to the Preferred Reporting Items for Systematic Reviews and Meta-Analyses guidelines for reporting systematic reviews.

### Eligibility criteria

To be eligible, studies were required 1) to be focussed on JIA-U; 2) to report data on patients with disease onset at or before age 16 years; 3) to report measures or indicators of the disease history of JIA-U, including risk/prognostic factors and treatment efficacy; 4) to be published in English. The exclusion criteria were: 1) studies focused on adults; 2) studies from which it has not be possible to extract data on children; 3) studies not focused on JIA-U; and 4) individual case reports. In order to overview and overall scan literature for the current used items regarding JIA-U, any additional selection of potential outcome measure indicators has been used. Any attempt to judge performance and reliability of each item has been considered into the selection procedure.

#### Information sources

Publications were retrieved using a computerized search of the following databases: EMBASE, Ovid MEDLINE, Evidence-Based Medicine (EBM) Reviews—ACP Journal Club, EBM Reviews—Cochrane Central Register of Controlled Trials, EBM Reviews—Cochrane Database of Systematic Reviews and EBM Reviews—Database of Abstracts of Reviews of effects. Publications between January 2000 and December 2018 were included.

#### Search strategy

Databases were searched with the key words “chronic uveitis” or “chronic iridocyclitis” or “recurrent uveitis” or “refractory uveitis” or “non-infectious uveitis” or “autoimmune uveitis” were crossed with “outcome” or “arthritis”. This strategy excluded records related to infectious and/or suppurative uveitis. Initially we did not include age limits in the search as MeSH terms in order to have the possibility to extract a sub-cohort of children from studies including both children and adults. No limitation with regard to the type of the study was entered. Further, relevant studies captured from the research strategy but focused on adults and/or not including children were excluded.

## Results

**Fig. 1:**
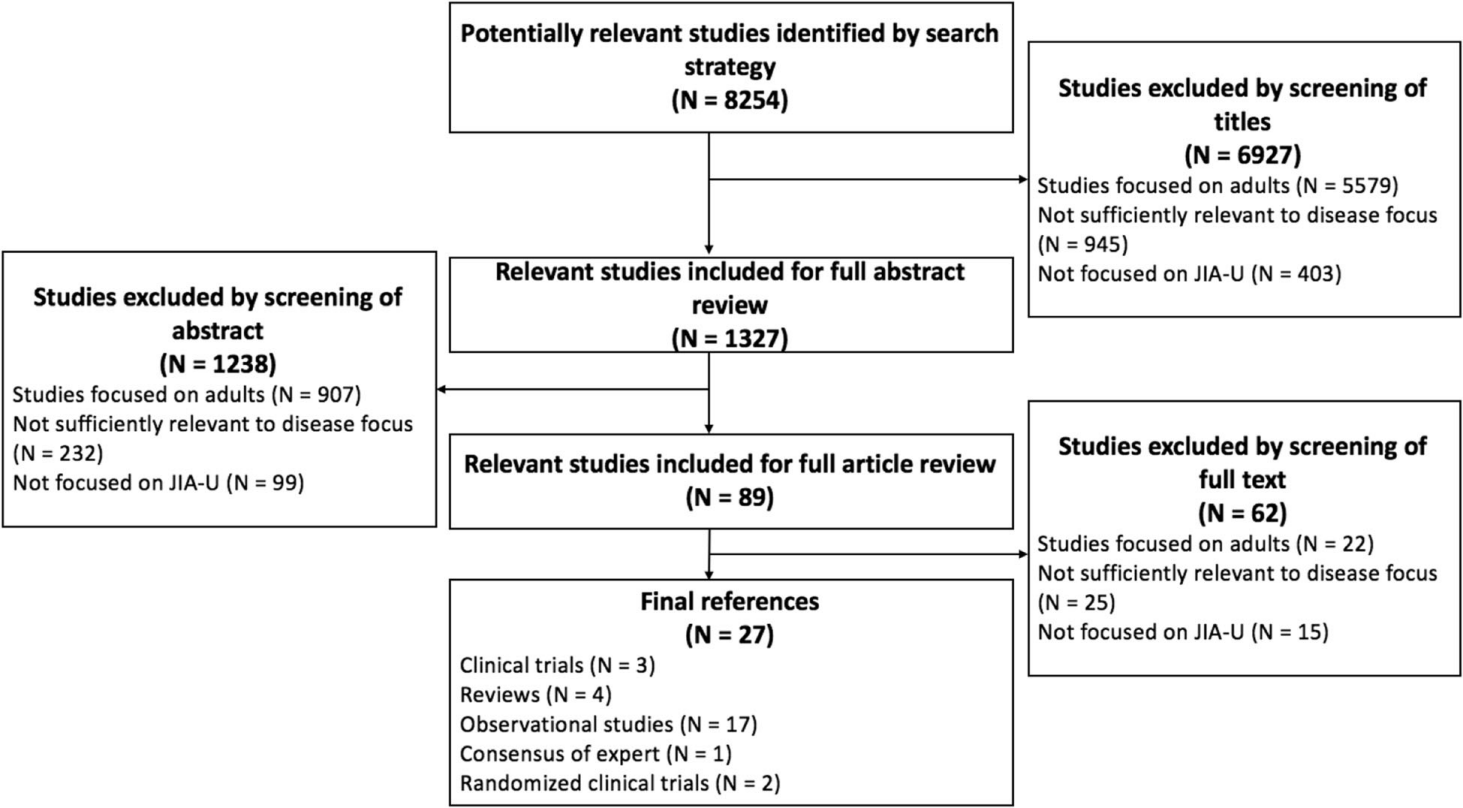
Study selection process flow diagram of literature research. Legend. N = number of identified studies

A total of 8254 articles were identified by searches of databases, and, from these the majority were excluded following examination of their titles and abstracts. Full text of the remaining 89 studies was analysed. From the selection process, a total of 27 relevant articles, including two randomized clinical trials (RCTs), were deemed eligible. A flow diagram of the study selection process, also reporting the reasons of the exclusion, is shown in Fig. 1. Among these studies we identified 12 outcome measures for use in JIA-U. Because of the different study design, we reported RCT data separately from the aggregate analysis of the other types of the studies.

The proposed outcome measures were:

- Grade of cells in the anterior chamber [5–13]
- Grade of flare in the anterior chamber [5, 7, 8, 12, 14, 15]
- Visual acuity [5, 7–13, 15–20]
- Amblyopia [5]
- Development of structural complications included: synechiae formation, cataract formation, macular edema epiretinal membrane formation, band keratopathy, ocular hypertension and glaucoma, ocular hypotony, vitreous haze and cells intraocular pressure abnormalities [5–12, 14–22]
- Use and sparing of oral corticosteroids and other immunosuppressive drugs [5, 7, 8, 10, 11, 13, 16, 17, 20]
- Surgery requirement [5, 8, 16, 18–20]
- Biomarkers [5–8, 14, 15, 21, 23–26]
- Bilateral disease [7, 8, 17–19]
- JIA persistence [7, 8]
- Quality of life [27–29]
- Uveitis subtype [20]

### Grade of cell in anterior chamber

Grading of the cell content in anterior chamber was considered a measure to assess uveitis activity [5–13]. Holland et al. noted that elevated cells, elevated flare, keratic precipitates, signs of intermediate uveitis and papillitis predicted future complications with baseline cells > 1 producing the greatest risk (RR: 16.06; 95% CI: 2.21–116.9; *p* = 0.006) [30]. It was demonstrated [7] that the risk of developing an ocular complication in an eye during follow up was increased by four-to-five fold if the eye had active anterior chamber (> 1 + cells) inflammation at presentation (RR = 5.06; *p* = 0.02 for affected eyes and RR = 4.20; *p* = 0.001 for better-seeing eyes). Woreta et al. [8] also showed that the presence of anterior chamber flare was a statistically significant risk factor for having at least one ocular complication at presentation of the disease. Presently, there is no evidence of a different risk attached to these 3 levels of activity [7], and some studies have shown no prognostic significance for cell counts in the presence of flare [31].

### Grade of flare in anterior chamber

Anterior chamber (AC) flare has been used as a measure either for the uveitis activity and the tissue damage [5]. Higher values are correlated with the presence of AC cells, usually during uveitis exacerbation [5]; however, flare might occur without cells in the AC [5] and persist despite of disease’s remission. Thorne at al. [7] suggested that anterior chamber flare > 1 was associated with a statistically significant increased risk of loss of visual acuity of 20/50 or worse. The same result was confirmed by other studies [8, 14, 15]. The prognostic significance of flare may be dependent on both coexisting structural damage and the effect of the treatment administered at the time of measurement [5]. Despite these uncertainties, the level of AC flare may be a good indicator of prognosis and a marker for damage of intraocular structures. For the detection of AC flare, the slit lamp is widely used; however, flare estimates may differ between observers and visits. Laser flare photometry is an objective technique, but since it is time consuming, expensive, and not feasible as a measure in many patients, Heiligenhaus et al. [5] considered that photometry should not be a necessary instrument, but remain a research tool for prospective RCTs.

### Visual acuity

Frequencies of poor visual acuity at presentation were assessed. Visual acuity (VA) cut-offs of 20/50 or worse (low vision) and 20/200 or worse (legal blindness) were used according to recommendation from the SUN Working Group [4]. In all analyzed studies [5, 7–13, 15–20], visual acuity was recorded as Snellen equivalents and were transformed to logarithm of the minimum angle of resolution score (logMAR) to facilitate the statistical analysis. Despite the majority of the studies [7–13, 15–20] have identified visual acuity as the main outcome measures for use in JIA-U, Heiligenhaus et al. [5] considered that visual acuity is not a useful measurement of uveitis activity; however, since it is influenced by prevalent complications, it is a very important indicator of ocular damage related to disease.

### Amblyopia

Only one article [5] considers amblyopia as a hypothetic outcome measures. Nonetheless its significant contributor to visual function outcomes and treatment costs, they authors conclude that it is not a useful measurement of uveitis activity or severity because its incidence is age dependent.

### Structural complications

Structural complications are the most common outcome measures used among the elegible articles [5–12, 14–22] and they were statistically associated with VA to the < 20/50 and 20/200 thresholds in all these studies. Indeed, structural damage is an important outcome measure that reflects both previous and current disease activity. While the development of new complications may reflect disease activity, they may also occur in the absence of active uveitis (e.g. epiretinal membrane formation, band keratopathy, ocular hypotony and hypertension, and glaucoma) [5]. They included: band keratopathy, cataract, synechiae, epiretinal membrane formation, macular edema, abnormal ocular hypertension and glaucoma or ocular hypotony, vitreous haze and cells. The most common causes for developing 20/200 or worse vision were mostly reported as cataract and band keratopathy in the visual axis. Cataract was defined as the presence of 1+ nuclear sclerosis or 1+ cortical change or trace posterior sub-capsular changes. Macular oedema (ME) was defined as the presence of macular thickening with or without cyst formation that was seen by clinical examination and / or spectral domain optical coherence tomography (OCT).

Recently the use of OCT has been proposed as an objective instrument-based measure of disease activity [22]. Most of the measures of disease activity are largely based on subjective clinical estimation with poor discrimination and accuracy. The universal adoption of high-resolution OCT has furnished a way of quantifying disease stages through objective instrument-based measurements. OCT provides non-invasive, repeatable, fast and well-tolerated measures that are sensitive to change in important disease processes (i.e. the measurement of central macular thickness (CMT) to measure severity of ME. The presence of ME has been reported by Taylor et al. [32] to be associated with both worse visual acuity and worse overall visual field sensitivity; moreover the qualitative differences in edema distribution were associated with differential impact on vision. Furthermore, Sugar et al. [33] demonstrated that each 100-lm reduction in CMT equated to a 6.5 letter increase in VA. A sensitivity analysis suggested that a reduction of 20% in macular thickness might be used as clinically valid improvement in VA (sensitivity 77%, specificity 75% for 10 letter improvement) [22]. Other studies have also associated other functional outcomes (i.e. reading VA, reading speed, and central retinal sensitivity) to OCT changes [34, 35]. Although CMT is the most worldwide used single OCT-derived measure of macular status, we have to admit that it is not the only parameter applicable It has been proposed OCT quantification of AC cells [36–39] and vitreous inflammation [22].

Ocular hypertension was defined as intraocular pressure (IOP) elevation > 21 mmHg and hypotony was defined as a IOP < 5 mmHg. Heiligenhaus et al. [5] suggested that elevated IOP and a glaucomatous optic disc should be reported as separate outcome measures. The presence of band keratopathy and posterior synechiae were diagnosed by slit-lamp examination. Epiretinal membrane formation and optic disk edema were diagnosed by indirect ophthalmoscopy and spectral domain OCT.

### Use and sparing of oral corticosteroids, nsaids and other immunosoppressive drugs

In adults, the SUN Working Group reported that reduction in dosages of prednisone to 10 mg/day or less while maintaining inactive uveitis should be considered the primary outcome for successful corticosteroid sparing. In children, there is no consensus whether it should be defined as for a desirable outcome measure, probably due to the well-know side effects related to a chronic administration of steroids, systemic rather than topic. Overall, some of the studies that we analyzed [5, 7, 8, 10, 11, 13, 16, 17, 20] have considered the use of corticosteroids and other immunosuppressive drugs as a possible outcome measure. On one hand, in a multivariate analysis [7] controlling for concomitant use of prednisone, use of immunosuppressive drug therapy was associated with a 74% reduction in the development of hypotony (RR = 0.26, 95% CI: 0.11, 0.59, *p* = 0.002), a 86% reduction in the development of epiretinal membrane (RR = 0.14; 95% CI: 0.02, 0.99, *p* = 0.05), and a 60% reduction in the development of 20/200 or worse visual acuity (RR = 0.40, 95% CI: 0.12, 0.98, *p* = 0.04). Additionally, previous use of corticosteroids or methotrexate was not confirmed as risk factors for ocular complication at presentation in multivariate-analysis [8]. However, data analysis, accounting for treatment variation over time, would be necessary for evaluating the association between use of immunosuppressive drugs and oral corticosteroid therapy and the development of ocular complications and visual loss [7].

### Surgery

Patients with JIA-U frequently require cataract surgery, vitrectomy, and glaucoma surgery [5, 8, 16, 18–20]. Cataract extraction with or without implantation of an intraocular lens was the most frequent procedure, followed by pars plana vitrectomy and glaucoma filtering surgery [16]. It was shown [8] that a history of intraocular surgery before presentation was significantly associated with 20/50 or worse and 20/200 or worse vision. Sabri et al. in a retrospective chart review of 1081 patients under 18 years of age with uveitis [20], also demonstrated that surgery was the single most important risk factor for reduced visual acuity at the last follow-up (*p* = 0.0086). However, in this study, the total number of surgeries, the types of surgery and the time interval from uveitis onset to the first surgery were not statistically significant risk factors for a reduced visual acuity outcome. Heiligenhaus et al. [5] suggested that surgery is not a primary outcome measure because indications for surgery and the surgical methods used are biased by the practice patterns of the treating ophthalmologist. Moreover, surgery might induce further inflammation of the eye, therefore, it may confound activity outcome studies. Despite this any surgery at baseline and/or after study entry should be documented as an indicator of severity of disease and tissue damage.

### Biomarkers

The main part of the studies considered antinuclear antibody (ANA), tested using HEP-2 cells, as an outcome measure of uveitis [5–8, 14, 15, 21]. Other biologic predictors aside from the ANA have been evaluated [6, 24–26]: anti-histone antibodies [6], A2-globulin [24], Interleukine 17A [25], Transthyretin (TTR) [26]. Although ANA has been noted to be a risk factor for uveitis development, its role as a predictor of uveitis complication is unknown. On one hand, Thorne et al. [7] and Woreta et al. [8] demonstrated that ANA positivity was associated with an increased risk of loss of visual acuity. Vitale et al. [14] also suggested an association between ANA titer and secondary glaucoma. Moreover, in a retrospective cohort study including 69 children, Parioli et al. have shown that positive ANA was associated with the 20/200 or worse threshold (*p* = 0.04; HR 1.0; 95% CI 0.4–2.3). On the other hand, Campanilho- Marques et al. [23] suggested that the presence of positive ANA does not represent a predictor of uveitis severity and does not have any correlation with the recurrence of either idiopathic anterior uveitis or JIA-related uveitis and cannot be used as a marker to predict the clinical course of ocular inflammation. Regarding other biologic predictors, Nordal et al. discuss the utility of anti-histone antibodies as predictors of uveitis with a level of IgM/IgG > 8 U /ml being significant [6]. Zulian et al. examined the role of A2-globuline and found a significant association with severe uveitis course [24]. Jawad et al. [25] demonstrated that serum IL 17 A levels are elevated in uveitis patients, particularly in active uveitis and it suggested that it should be used as marker of disease activity. Ayuso et al. [26] investigated the presence of biomarkers in aqueous humor from 116 children with JIA associated uveitis and they found out that Transthyretin (TTR) was a potential intraocular biomarker of the disease. However the role of these markers in the proof of disease activity needs further investigation.

### Bilateral disease

Dana et al. [17] through a retrospective study of 43 patients found that the odds of better vision in at least one eye (and hence better overall functional vision) in patients with bilateral disease are two to one. The odds of visual improvement in a diseased eye are higher in unilateral cases, in those who experienced visual improvement (8/10 patients). Other studies [7, 8] have shown that bilateral uveitis was a statistically significant risk factor for having at least one ocular complication at presentation. Gregory et al. [18] demonstrated that bilateral disease was associated with an increased risk of developing < 20/50 vision during follow up. Edelsen et al. confirmed the same results [19]. Bilateral disease could eventually be considered an important outcome measure even though further studies should be carried out, it presents more severity than activity.

### Persistent oligoarthritis JIA

A retrospective study of 75 patients with JIA associated uveitis [8] suggested that persistent oligoarthritis type of JIA was a statistically significant risk factor of having at least one ocular complication at presentation. Further studies would be necessary to assess if it could be a useful measurement of uveitis activity or severity.

### Quality of life

Quality of life (QOL) is known to be an important construct, particularly related to chronic disease [29]. Vision-related QOL (VRQOL) is related to, but not identical to, visual function. VRQOL represents the degree to which vision impacts an individual’s ability to complete activities of daily living and one’s social, emotional, and economic well-being. VRQOL can be assessed by measuring the degree of impairment experienced in activities of daily living that rely on sight (i.e., impaired daily function secondary to visual difficulties is a proxy for visual function) [27]. Before Angeles-Han study [27] there was a paucity of instruments that measure visual disability and vision-related QOL (VRQOL) in children (i.e. CHAQ-Childhood Health Assessment Questionnaire, PedsQL-Pediatric QOL inventory) and none were specific for children with uveitis that would make the assessment of QOL related to visual disability difficult in this population. The Effects of Youngsters’ Eyesight on Quality of Life (EYE-Q) is a patient-based self-report that consists of 23 items for children ages 8–15 years, and 26 items (3 additional items on driving) for children ages 16–18 years. This study provided evidence of the validity and reliability of the EYE-Q in the measurement of VRQOL. Moreover in a subsequent study [28] EYE-Q was modified with uveitis specific items and it was compared with CHAQ and PedsQL. EYE-Q appeared to be a valid and reliable measure of vision related function in pediatric uveitis in children 5–18 years of ages. However it was discovered that several items in EYE-Q were inapplicable to children younger than 5 years old since many tasks were school specific or required independent skills. Therefore a module for non-school aged children is needed. Meanwhile the use of the Children Visual Function Questionnaire (CVFQ) is suggested, it measures visual function through parent reports in children < 7 years old, though is not disease specific. Despite a larger cohort study is needed for further validations, EYE-Q might be an important tool in the assessment of visual outcome in childhood uveitis and an improvement over general measures in detecting changes in vision-related function.

### Uveitis subtypes

Uveitis is also classified by course in acute, recurrent or chronic. Disease duration that exceeds 3 months is generally considered “chronic”, as opposed to “acute” disease, which typically comes on quickly and lasts less than 6 weeks. Recurrent uveitis is defined as repeated episodes of uveitis separated by periods of inactivity without treatment of ≥3 months in duration. [4]. Given that, uveitis subtypes are: chronic anterior (the most common), acute anterior, recurrent anterior and panuveitis. A retrospective chart review of 1081 children with JIA-U [20], revealed that the development of complications (*p* < 0.0001) and a blind visual outcome (*p* = 0.0096) was associated with the type of uveitis. Furthermore blindness occurred within 1 year of diagnosis of uveitis in 5/10 (50%) cases. Indeed, among the 175 eyes analysed, while over 90% of eyes with anterior acute uveitis, anterior chronic uveitis and anterior recurrent uveitis had a good visual outcome, over 80% of eyes with panuveitis had a blind visual outcome (Cox regression, *p* < 0.0001). Type of uveitis (especially panuveitis), considering these results, could be evaluated as a possible outcome measurement as well, even though further studies are needed.

### Aggregate analysis

Figure 1 reports in final reference the type of selected studies, including the 2 RCTs.

Among the non-RCT included studies, 44% (11/25) included one or more variables related to disease activity as primary outcome measures; 56% (14/25) included visual acuity as a primary outcome measure; 68% (17/25) included one or more variables of disease-associated tissue damage or complications as primary outcome measures; 24% (6/25) included disease features as a primary outcome measure; 44% (11/25) included laboratory features as primary outcome measure; 8% (2/25) included JIA features as a primary outcome measure; 12% (3/25) included quality of life as a primary outcome measure; 44% (11/25) included management as a primary outcome measure.

### Outcome measures in RCTs

The present systematic review was able to identify 2 RCTs, both aiming to prove Adalimumab efficacy in JIA-U: SYCAMORE [40] and ADJUVITE [41] trials.

The first one concluded that Adalimumab is associated with a lower rate of treatment failure than placebo among children and adolescents with active JIA-U who were taking a stable dose of MTX. Sycamore trial addressed its purpose defining the primary endpoint -time to treatment failure- by the use of the SUN cell-activity score. The grade of cells in the anterior chamber under a slit-lamp examination was therefore the principal outcome measure used. Secondary end points included as additional outcome measures the use of topical and systemic glucocorticoids, and health-related quality of life instruments, as Childhood Health Assessment Questionnaire and the Child Health Questionnaire [40].

ADJUVITE trial resulted in favour of using Adalimumab in patients with early onset, chronic anterior uveitis, which is in most cases associated with JIA, in case of inadequate response to topical therapy and MTX. The primary outcome was response to treatment, defined as a reduction of at least 30% of ocular inflammation quantified by laser flare photometry without worsening of cell counts or protein flare on slit-lamp examination according to SUN criteria. Secondary outcomes included modifications of the dose of topical and/or systemic steroid therapy over the trial [41].

## Discussion

The aim of this study was to review the available evidence regarding outcome measures for JIA-U. Assessment of childhood JIA-U needs urgently to develop better outcome measures, as trial endpoints in clinical studies for development and licensing drug, as well as in driving treatment decisions in routine clinical practice. Nonetheless any attempt to judge the quality and the performance of the reported outcome measures has been acted, the present systematic review provides evidence that paediatric outcomes in JIA-U are particularly scarce and heterogeneous [42]. Twelve outcome measures with distinct dimensions have been identified: disease activity (i.e. grade of flare, grade of cells); visual function performance (i.e. visual acuity); disease-associated tissue damage or complications (i.e. cataract, amblyopia); disease features (i.e. bilateral disease; uveitis subtype); laboratory features (i.e. biomarkers); JIA features (i.e. persistence of disease); quality of life (i.e. EYE-Q); management (i.e. use and sparing of oral corticosteroids and other immunosuppressive drugs; surgery requirement). It may be argued that the different distribution of the different entered outcome measures and dimensions may reflect a different point of view of the clinician, making treatment decisions primarily based on disease activity, visual acuity and disease damage. The impact of the disease on function and quality of life, probably the primary concern of parents and/or patients, seems indeed quite fare and neglected. Surely, all dimensions are inter-related, but the relationship among them is quite complex and mostly it may be hard to weight each one of them independently. For example, worsening visual acuity is associated with worsening patient-reported visual function, but the relationship is not necessarily direct, and the related correlation not really true.

Disease-associated tissue damage with and/or without complications is the most common used measure of disease activity, and as primary outcome measure is statistically associated with VA to the < 20/50 and 20/200 thresholds. Additionally, since structural damage may reflect previous and current disease activity, it can be considered a suitable candidate as complete outcome measure.

Current measures of disease activity are largely based on subjective clinical estimation. Of note, there are profound prognostic differences between the absence of cells and the presence of even a small number. Additionaly, since most clinical activity in JIA-U occurs within 3 grades of activity (0.5–2), aggregated counts may also lose sensitivity [5]. The development of quantitative imaging in uveitis in adults is most established using OCT measurement of CMT to measure severity of ME [22]. Uveitic ME is undoubtedly an outcome measure to be considered: it is the single most important reversible cause of sight-loss, and, since responsive to treatment, it can be the objective focus of most clinical trials. Additionally, ME measurement by OCT is the prototypic demonstration of quantitative imaging in uveitis. However the use of uveitis quantitative imaging, and their development as endpoints, is still in a relatively early phase. Indeed, in the description of a patient with ME, central macular thickness is only one feature; it does not aim to be a comprehensive assessment of the functional, psychological, or societal consequences of uveitis. Even so, it is an extremely useful parameter, which let us assess severity, progression and response to treatment at a tissue level in an objective way, which was never possible before the OCT use. In order to get the most objective and comparable measurement as possible, when clinical clues of ME are present, we suggest obtaining OCT imaging even in children [11, 12]. Despite the need of further investigations, ME might be used, by itself, as uveitis outcome index apart from the other complications. Quantitative imaging approaches are now being developed and validated for other key inflammatory parameters such as anterior chamber cells, vitreous haze, retino-vascular leakage, and chorioretinal infiltrates [36–39]. The development, validation, and adoption of sensitive and discriminatory measures of disease activity are an unmet need since its tremendous implications in drug development and routine clinical care. Interestingly, in adults, OCT-derived parameters have become standard outcome measures in clinic faster than they have become acceptable as trial endpoints by the regulatory authorities. Therefore, FDA have pointed out that while OCT-derived endpoints are ‘well-defined and reliable’ they do not directly measure how a patient ‘survives, feels or functions’.

Regarding VA, the majority of the studies [7–13, 15–20] have identified VA as the main outcome measure for use in JIA-U. However, due its strong relationship with present and past ocular damage [5], it remains a significant indicator of related disease eye damage, whilst its reliability as measure of activity remains debated. The assessment of uveitis activity by visual acuity its self, without data from slit lamp evaluation, seems inappropriate. Change over time in visual acuity, measured in LogMAR, mostly before and after treatment, seems indeed a more reliable method to assess uveitis activity regarding to visual acuity.

Visual function assessment, appropriately adjusted for age, relevant activities and reporting skills [22, 27], can expand the utility of quality of life measures. The impact of altered visual function on quality of life may be objectified through the EYE-Q, an uveitis-related quality of life assessment questionnaires specific for children. Before Angeles-Han study [27], such a paediatric-customized assessment was not available. This and a subsequent related study [28] make this tool a disease-specific and universally agreed upon outcomes, that leads to reduce selective reporting and reporting bias.

There is no consensus whether the reduction of topical and systemic corticosteroids should be defined as desirable outcome measure. Despite this, many studies [5, 7, 8, 10, 11, 13, 16, 17, 20] considered it as one of them. Of note, both the available RCTs entered this information, even though used as secondary outcome [40, 41]. As matter of fact, tapering/stopping steroids, particularly systemic, represents an indirect measure of activity and good control of disease. However, we suggest that it is important information to be collected in trials, even if furthermore data analysis would be necessary.

Additionally, surgery may not be a primary outcome measure since indications for surgery and surgical methods used are definitively biased by the practice patterns of the treating ophthalmologist.

At the present, there is no consensus either about biomarker role, as outcome measure. Aside from ANA, the other biomarkers resulted from this systematic review have not been duplicated in larger and multicenter cohorts, thus limiting overall their potential role. Of course, their use in clinical practice as outcome measure seems currently not applicable. ANA may reflect a risk for a more severe course, but it does not seem a sensible outcome measure, related to a change over treatment. Further investigation are needed to proof the other mentioned biomarkers as part in the disease activity.

The development of complications and a blind visual outcome seems to be associated with the type of uveitis, especially panuveitis [20] and bilateral disease [7, 8, 17–19]. Therefore it can be suggested that type and disease features may be part of a composite outcome measures, but again the type of uveitis reflects the severity and not the activity of the disease. Persistent JIA oligo-arthritis results a statistically significant risk factor of having ocular complications [8], but further studies would be necessary to assess if it could be a useful measurement of uveitis activity or severity.

While our systematic review does not attempt to provide the solution to overcome the heterogeneity in uveitis studies, it does provide an estimate of the scale of the problems and provides data to inform this important debate. The broad variation in outcome measures chosen by the investigators of eligible analysed articles is, in itself, an indicator that there is likely to be no easy answer to this issue. At the present, the strongest evidence available in treating JIA-U comes from SYCAMORE and ADJUVITE trials. However, as the broad heterogeneity reported in the rest of eligible studies, they failed to use the same measure for the primary end-point. Nonetheless achieving the same conclusions, thus making tremendous step-forwards in JIA-U clinical setting, they used different instruments –slit lamp versus laser flare photometry- to assess and measure, apparently, the same outcome variable of inflammation. In our opinion, this fact represents the paradigm of the need to harmonize the choice of the outcome measures.

Considering a composite score, we would however, underscore the potential risk that one may fail to detect therapeutic benefit due to the high level of “noise” introduced by joining in one unique score a too-wide range of clinical entities. Considering the “composite” feature of an outcome measure, while it may be argued that such outcome measures provide a comprehensive “holistic” assessment of the patient state, frequently including visual acuity, it is likely that the frequent use of composite measures in these series of reviewed is just simply driven by the lack of a single outcome measure suitable for all patients. In other words, lacking something better, of necessity, virtue.

In adults, it has been tackled the issue of heterogeneous outcome measures in clinical trials by establishing “core outcome sets” (COS). This approach provides a standardized set of outcome measures that are reported in all clinical trials of a condition under consideration, while still allowing the investigator discretion to choose his or her own primary or secondary outcome measures [43]. The use of COS may enhance evidence synthesis by reducing heterogeneity, outcome-reporting bias and improving the statistical power of any meta-analysis. COS development is supported by a number of initiatives, such as COMET (Core Outcome Measures in Effectiveness Trials) and has been endorsed by the Cochrane Library, the GRADE (Grading of Recommendations Assessment, Development and Evaluation) working group, and the WHO [43, 44]. In summary our systematic review formally surveys the heterogeneity present around outcome measures in recent and current articles related to JIA-U in children.

According the OMERACT filter criteria [45], probably, none of the 12 selected outcomes, alone, can fulfil all the required variables of validity. Surgery, and structural complications represent more risk factors of worst outcome rather than a reliable and feasible measure. Oligo-arthiritis persistent JIA is of course a risk factors but not an outcome measure. Grade of cells or grade of flare, probably the most “true” outcome measure for active disease, are, however, lacking of discrimination and reliability, since they can differ in repeated measures, and may change if performed by different ophthalmologists. Visual acuity might be convergent with the other variables, but it cannot be really sensitive to change, since it cannot be responsive to small significant clinical changes. In addition, its reliability, as stability when measured repeatedly, can be affected in children. QOL, as any childhood measures of this type, require data acquisition on disease impact from parents and from caregivers and patients, both. Therefore it appears that multiple and composite domains seem necessary to capture all aspects of such complicating, sight and visual-threatening a disease.

## Conclusions

Our systematic review surveys the broad heterogeneity of JIA-U outcome measures, even in RCTs. It provides data, describing the dimension of the debate. Our analysis did not attempt to judge the reliability of the reported or proposed outcome measures; additionally we did not include specific limitations and qualitative assessment for selection procedure of the entered studies and their related outcome measures. Nonetheless the number of current outcome measures for JIA-U results extremely fair. We argue that the challenging issue of outcome measure selection for clinical trials and clinical care of JIA-uveitis children needs to be addressed, physicians dealing with uveitis need to work, in a multidisciplinary way, towards a new consensus regarding a common approach to identify suitable and efficient outcome measures in JIA-uveitis. A composite outcome measure, including activity and damage measures, might be a reliable method to capture and measure a such challenging disease as uveitis. The Multinational Interdisciplinary Working Group for Uveitis in Childhood (MIWGUC) is currently pursuing this aim and its effort represents a first additional step toward this aim.

## Data Availability

Not applicable.

## Abbreviations

AC: Anterior chamber
ANA: Antinuclear antibody
CHAQ: Childhood Health Assessment Questionnaire
CMT: Central macular thickness
COMET: Core Outcome Measures in Effectiveness Trials
COS: Core outcome sets
CVFQ: Children Visual Function Questionnaire
EYE-Q: Effects of Youngsters’ Eyesight on Quality of Life
FDA: Food and Drug Administration
GRADE: Grading of Recommendations Assessment, Development and Evaluation
IOP: Intraocular pressure
JIA-U: Uveitis associated to Juvenile Idiopathic Arthritis
ME: Macular edema
OCT: Optical coherence tomography
OMERACT: Outcome Measures in Rheumatology
PedsQL: Pediatric QOL inventory
QOL: Quality of life
RCTs: Randomized controlled trials
SUN: The Standardization of Uveitis Nomenclature
TTR: Transthvretin
VA: Visual acuity
VRQOL: Vision-related QOL
WHO: World Health Organization
